# Continuous low water pressure dissection technique minimizing air bubbles during underwater endoscopic submucosal dissection

**DOI:** 10.1055/a-2368-9612

**Published:** 2024-08-07

**Authors:** Mitsuru Nagata

**Affiliations:** 1 Department of Endoscopy, Shonan Fujisawa Tokushukai Hospital, Fujisawa, Japan

Underwater endoscopic submucosal dissection (UESD) in saline offers several advantages 1 2 . However, air bubbles generated during UESD can impair the visual field, particularly when they accumulate in the hood. To address this issue, outlets can be introduced on the side of the hood to facilitate air bubble removal 3 4 . Nevertheless, air bubbles accumulate in the hood in cases where the lesion is located opposite to gravity, the tissue obstructs the air bubble outlet, or large air bubbles form. This repeatedly necessitates the use of the waterjet function of the endoscope to flush out the air bubbles. Under saline, air bubbles are generated by the evaporation of saline caused by the energization of the electrosurgical knife, which creates a “vapor pocket” that enables the knife to perform dissection 5 . Continuous high water pressure can make dissection difficult by hindering the formation of a sufficient vapor pocket, whereas continuous low water pressure helps maintain dissection power while minimizing and removing air bubbles. Therefore, we propose the continuous low water pressure dissection technique (CDT), which helps maintain the visual field during UESD ( Fig. 1 ).

**Fig. 1:**
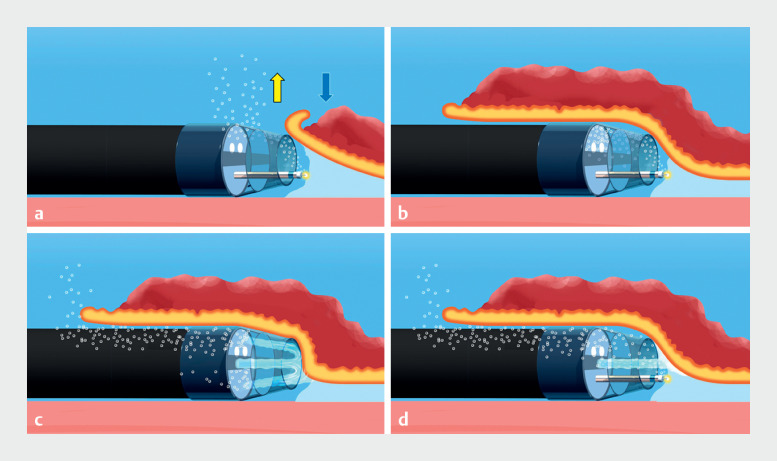
Comparison of conventional dissection and continuous low water pressure dissection techniques using a tapered hood with air bubble outlets in underwater endoscopic submucosal dissection. **a** Buoyancy (yellow arrow) automatically removes air bubbles through the air bubble outlets when the lesion is on the gravity side (blue arrow). **b** If the lesion is located opposite to gravity, the tissue obstructs the air bubble outlet, or large air bubbles form, and air bubbles accumulate in the hood. **c** Backflow of water pressure using the waterjet can remove air bubbles through the air bubble outlets. **d** Continuous low water pressure dissection technique. Dissection combined with continuous low water pressure using the waterjet function of the endoscope minimizes and removes air bubbles without the need to turn the waterjet on and off.

Video 1 demonstrates UESD using a hood (ST hood short type; DH-29CR; Fujifilm, Tokyo, Japan) with self-made air bubble outlets for a colorectal tumor. As half of the lesion was located against gravity, air bubbles accumulated in the hood. The flushing pump setting (OFP-2; Olympus, Tokyo, Japan) was adjusted from strong to weak after creation of the mucosal flap, and CDT was initiated. CDT removed air bubbles that entered the hood without the need to turn the waterjet on and off, thereby expediting the procedure. Without changing the output of the Swift Coagulation (Effect 3, 30 W) in the electrosurgical generator (VIO300D; Erbe, Tübingen Germany), dissection power was maintained ( Fig. 2 ).

Underwater endoscopic submucosal dissection using a continuous low water pressure dissection technique and a hood with four self-made air bubble outlets for resection of a laterally spreading tumor in the cecum.Video 1

**Fig. 2:**
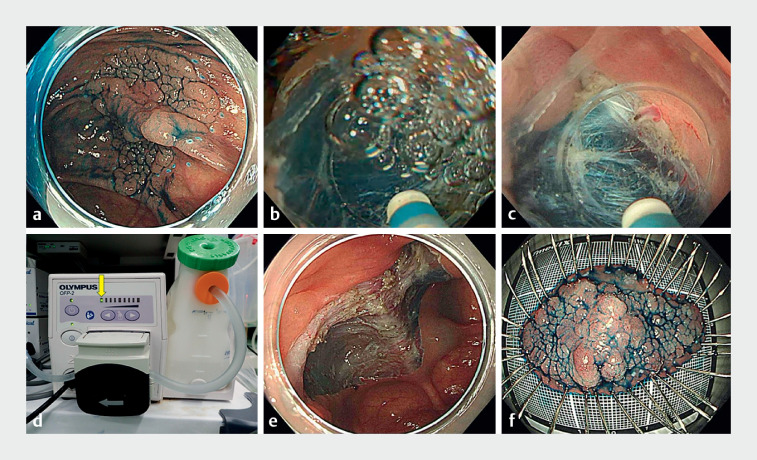
Underwater endoscopic submucosal dissection using the continuous low water pressure dissection technique and a tapered hood with air bubble outlets. **a** The laterally spreading tumor in the cecum was sprayed with indigo carmine. **b** As half of the lesion was located opposite to gravity, air bubbles accumulated in the hood. **c** The continuous low water pressure dissection technique effectively minimized and removed the air bubbles. **d** The flushing pump output (OFP-2; Olympus, Tokyo, Japan) was adjusted to the weakest setting (yellow arrow) to maintain the appropriate water pressure for dissection power. **e** The lesion was resected en bloc without perforation. **f** The resected specimen was sprayed with indigo carmine. Pathological examination revealed high grade dysplasia (World Health Organization classification), measuring 60 mm in size, with negative lateral and vertical margins.

In conclusion, CDT helps maintain the visual field during UESD, enhancing the usefulness of a hood with air bubble outlets.

Endoscopy_UCTN_Code_TTT_1AQ_2AD_3AD

## Notes

Endoscopy E-Videos https://eref.thieme.de/e-videos
*E-Videos* is an open access online section of the journal *Endoscopy* , reporting on interesting cases and new techniques in gastroenterological endoscopy. All papers include a high-quality video and are published with a Creative Commons CC-BY license. Endoscopy E-Videos qualify for HINARI discounts and waivers and eligibility is automatically checked during the submission process. We grant 100% waivers to articles whose corresponding authors are based in Group A countries and 50% waivers to those who are based in Group B countries as classified by Research4Life (see: https://www.research4life.org/access/eligibility/ ). This section has its own submission website at https://mc.manuscriptcentral.com/e-videos .

## References

[1] Nagata M Usefulness of underwater endoscopic submucosal dissection in saline solution with a monopolar knife for colorectal tumors (with videos) Gastrointest Endosc 2018 87 1345 1353 10.1016/j.gie.2017.11.032 29242059

[2] Nagata M Underwater endoscopic submucosal dissection in saline solution using a bent-type knife for duodenal tumor VideoGIE 2018 3 375 377 10.1016/j.vgie.2018.09.015 30505999 PMC6251784

[3] Nagata M Tapered hood with wide holes in its sides for efficient air bubble removal during underwater endoscopic submucosal dissection Dig Endosc 2022 34 654 10.1111/den.14232 35000224

[4] Nagata M Underwater endoscopic submucosal dissection using a tapered hood with air bubble outlets for a subcircumferential duodenal tumor Dig Endosc 2024 36 225 227 10.1111/den.14696 37830130

[5] Botto H Lebret T Barré P Electrovaporization of the prostate with the Gyrus device J Endourol 2001 15 313 316 11339400 10.1089/089277901750161917

